# Retraction: Allopregnanolone restores the tyrosine hydroxylase‐positive neurons and motor performance in a 6‐OHDA‐injected mouse model

**DOI:** 10.1111/cns.13780

**Published:** 2021-12-17

**Authors:** Zhi‐Chi Chen, Tong‐Tong Wang, Wei Bian, Xin Ye, Meng‐Yi Li, Juan‐Juan Du, Peng Zhou, Huai‐Rui Cui, Yu‐Qiang Ding, Yan‐hua Ren, Shuang‐Shuang Qi, Yuan‐Yuan Yuan, Min Liao, Chen‐You Sun

**Affiliations:** ^1^ Department of Anatomy School of Basic Medical Sciences Wenzhou Medical University Wenzhou China; ^2^ Institute of Neuroscience School of Basic Medical Sciences Wenzhou Medical University Wenzhou China; ^3^ Department of Histology and Embryology School of Basic Medical Sciences Wenzhou Medical University Wenzhou China; ^4^ Department of Pharmacy Second Affiliated Hospital Wenzhou Medical University Wenzhou China; ^5^ School of Basic Medical Sciences Zhejiang Chinese Medical University Hangzhou China

Retraction: Chen, Z‐C, Wang, T‐T, Bian, W, et al. Allopregnanolone restores the tyrosine hydroxylase‐positive neurons and motor performance in a 6‐OHDA‐injected mouse model. CNS Neurosci Ther. 2020; 26: 1069–1082. https://doi.org/10.1111/cns.13432. The above article, published online on 30 June 2020 in Wiley Online Library (wileyonlinelibrary.com), has been retracted by agreement between the authors, the journal's Editor in Chief, Dr Jun Chen, and John Wiley & Sons Ltd. The retraction has been agreed following concerns raised about possible image manipulation in Figures 5A, 6C, and 6H. The concerns were shared with the authors of the article who confirmed that some of the bands had been manipulated during figure preparation. As a result, the Editor‐in‐Chief no longer has confidence in the results and conclusions drawn and the article is being retracted.

